# Comparison of acetylcholinesterase among employees based on job positions and personal protective equipment in fuel station

**DOI:** 10.5620/eaht.2023018

**Published:** 2023-09-27

**Authors:** Chan Pattama Polyong, Anamai Thetkathuek

**Affiliations:** 1 Occupational Health and Safety Program, Faculty of Science and Technology, Bansomdejchaopraya Rajabhat University, Bangkok 10600, Thailand; 2 Department of Industrial Hygiene and Safety, Faculty of Public Health, Burapha University, Chonburi Province 20131, Thailand

**Keywords:** Serum AChE activity, Job positions, PPE, Fuel stations, employees

## Abstract

The purpose of this study was to compare the levels of acetylcholinesterase (AChE) among employees based on job positions and the wearing of personal protective equipment (PPE) in fuel station areas. The sample group consisted of 200 people sorted into various groups, including (i) Inside fuel dispenser area (I-FDA) group consisting of 100 employees for refueling, cashier, and loading fuel into storage tanks, and (ii) Outside fuel dispenser area (O-FDA) group, consisting of 100 employees working in convenience stores, food stores, coffee shops, and offices, as well as general staff and car washers. Data were collected using interview questionnaires and blood samples were analyzed for AChE activity. The results showed that the I-FDA group had a significantly lower mean of serum AChE (7.38±1.73 U/ml) than the O-FDA group (7.85±1.49 U/ml) (*p*<0.05). The I-FDA group had a 2.43 times higher risk of abnormal serum AChE than the O-FDA group (OR=2.43, 95%CI=1.05-5.60). When considering the risk factors for job positions, it was found that refueling and cashier positions had lower AChE activity levels than those who did not (*p*<0.05). In the part, employees for food sales in a closed building and those wearing PPE masks had significantly higher AChE activity levels than those who did not or who wore them (*p*<0.05). Based on the aforementioned, employers at fuel stations should strictly determine the appropriate measures for wearing a PPE mask. Further, employees should be provided accommodation away from oil supply or enclosed buildings during breaks to reduce the effects on the neurotransmitter.

## Introduction

Employees working in fuel station areas, both indoors and outdoors, are exposed to many chemicals, especially organic solvents (OS) [1] and a complex mixture of hydrocarbons, including volatile organic compounds (VOCs), such as benzene, and polycyclic aromatic hydrocarbons (PAHs), such as pyrene and benzo[a]pyrene [2]. These chemicals can enter the body through various channels, especially the respiratory system, which is the main route of exposure to the chemicals in fuel [3].

Occupational exposure to chemicals in fuel has been assessed between refuelers and convenience store employees at fuel stations. It was found that there was no difference in exposure to benzene [1]. Surprisingly, it was reported that convenience store employees were exposed to higher concentrations of toluene and xylene than fuel employees [4]. In Thailand, an exposure assessment of BTEX (Benzene, Toluene, Ethylbenzene, and Xylene) found that those working in fuel station areas had urine BTEX metabolized at 29 percent [5], exceeding the standards of the American Conference of Governmental Industrial Hygienists (ACGIH). It can be seen that OS is a contamination problem in various operating fuel station areas. There are many occupations in this area that can result in negative health effects [4,6].

The nervous system is the primary target organ for OS toxicity [7,8]. In the past, studies compared the neurological symptoms in the same person before and after working at a fuel station. It was found that work in the present had 2.17 times more neurological symptoms than in the past, before working at a fuel station [6]. This is because OS substances can pass through the blood-brain barrier into the nerves, and OS involves toxicity through the inhibition of AChE [9]. It has been reported that gasoline-sniffing syndrome changes the electroencephalogram, and slowed nerve conduction velocities [10]. Increasing severity can result in cognitive impairment, encephalitis, and death [11].

Cholinesterase enzymes are important for the body. They can be classified into two types: acetylcholinesterase (AChE) and butyrycholinesterase (BchE). AchE is found in red blood cells and nervous system tissue. It is responsible for the degradation of the neurotransmitter acetylcholine (Ach). The BchE type is found in the serum and extraneurotic tissue; it plays a role in the detoxification and hydrolysis of Ach [12]. A study between the two enzymes and OS susceptibility showed that AChE was more susceptible to changes than BChE. The AChE is an enzyme catalyzing the hydrolysis of acetylcholine, which is an important neurotransmitter for neurocognitive functions, into acetate and choline [13]. The mechanism of OS toxicity can inhibit AChE until the congestion of the neurotransmitter Ach and illness occurs [9].

The effect of OS exposure affects the inhibition of AChE, leading to the over-accumulation of the neurotransmitter acetylcholine in the cholinergic nerve synapses [14]. In vitro experiments, it was found that OS exposure affected the reduction of AChE in erythrocyte cells, and OS significantly decreased the acetylcholinesterase activity of the human erythrocyte membrane [15]. In addition, fuel inhalation caused a significant alteration in the monoamine neurotransmitter, involving the reduction of sodium-potassium pump (Na^+^/K^+^-ATPase), AChE and superoxide dismutase activities [16]. Studies on people exposed to an unknown mixture of chemical fumes showed that those exposed to the substance had decreased AChE levels and marked neurological symptoms including headache, dizziness, and decreased perception [17]. Therefore, AChE is a maker of effect and can be used to assess neurological disorders.

Job positions and wearing PPE are important risk factors that influence exposure to the substances in fuel and can affect health [18]. There have been studies that mentioned exposure in jobs such as refueling workers, convenience stores, food sales at stations, etc. No difference was found in exposure to OS [1,5], and health effects were reported in terms of neurological symptoms [6,18,19]. However, studies on the link between OS exposure and effects on AChE activity are lacking.

Regarding PPE factors, wearing PPE is usually for the purpose of preventing chemical exposure. However, Kapeleka et al. [20] stated that farmers wearing individual PPE did not affect AChE activity. Farmers can potentially be exposed to chemicals from aerosol sprays, which can enter the body through a variety of channels, though breathing and the skin are the main routes. Wearing only one type of PPE may not provide complete protection against pesticides, which is different from the fuel particles found in the form of vapor. Breathing is therefore the main route of exposure into the body. Therefore, careful attention to respiratory protection should be emphasized [1].

The health surveillance of neurotoxin exposure using biomarkers of effect from AChE activity in OS-exposed workers is an interesting alternative as AchE is an important indicator. However, the change in AChE activity is a marker of the sensitivity of the body to chemicals [21]. In Thailand, there is a law addressing the health surveillance of workers with health checks based on risk factors. It is a test of exposure and impact indicators. OS exposure to the nervous system is well known [17]. On the other hand, laboratory investigations with enzymes are involved in the nervous system; AChE is not recommended as a guide for risk factor assessment. There are also differences in the assessment of exposure to pesticides [22]. Therefore, increasing academic support may also result in the surveillance of AChE activity in OS-exposed individuals. This would lead to the prevention and reduction of health effects on more severe levels.

From the review of the literature, it can be seen that OS is still a problem at fuel stations as it affects the nervous systems of employees. In this study, health outcomes were investigated with AChE activity, an important neurotransmitter [13] and a sensitive and accurate marker [9], especially in those with low exposure to chemicals [23]. However, it has been used to assess the effects of pesticides mainly among farmers who have been exposed to organophosphates and carbamate insecticides [14,20]. Although some pesticides contain up to 34.6% substituted benzene [20], this was previously found in a laboratory study [9,15]. Therefore, the purpose of this study was to study the comparison of AChE activity in employees based on job positions and the wearing of PPE in fuel station areas. This field study aimed to identify the most at-risk job positions and explain how to protect oneself with the use of PPE, which will lead to more practical advice in the field.

## Materials and Methods

### Population and sampling

This pilot study examined AChE activity in a sample of fuel station operators from OS exposure, for whom AChE activity has not been reported previously. The calculation formula of Faul et al. [24] was used by the G*Power program to compare the difference between two means to determine the effect size of medium = 0.5 [25], α error prob = 0.05, power (1-β error prob) = 0.95. The sample size was two groups with 100 people per group, divided into (i) Inside fuel dispenser area (I-FDA) workers group of refuelers, cashiers, and people loading oil tanks, and (ii) Outside fuel dispenser area (O-FDA) workers group of people working in convenience stores, food shops, offices, car wash services, and general staff. Cluster sampling was technical because the operations at each fuel station were similar. Therefore, all stations were randomly selected until the number of samples was reached according to the sample size. The inclusion criteria were: having worked at a fuel service station for not less than 3 months, having no underlying liver or kidney disease, not suffering from alcoholism, not undergoing usage of medications for neurological or psychiatric diseases and being willing to voluntarily participate in the research. The exclusion criteria included employees who were sick or absent on the day of the sample collection.

### Human research ethics

This study followed Helsinki's declaration through the Human Ethics Review of the Institutional Review Board for Protection of Human Subjects in Research of Burapha University (BUU-IRB), Certificate No. 019/2020.

### Research tools and quality

The instruments used in this study were divided into 2 types: Interview questionnaire and blood collection analysis of AChE activity as follows:

1) The interview questionnaire consisted of 14 items divided into 4 parts: (i) General information for 6 items including gender, age, weight, height, educational status and income; (ii) Behavior for 3 items including alcohol drinking, smoking and taking drugs; (iii) Dietary in the past week for 10 items including eating fresh fried or leafy green vegetables, tomato, cauliflower, lettuce, long beans, fruit juice box, prunes or dried fruit, turmeric, honey, durian, and (iv) Work history for 5 items including job positions, experience working hours per day, working time per day, behavior of wearing personal protective equipment (PPE), and location of fuel station. The interview questionnaire was assessed for content validity by 3 occupational medicine and occupational health and safety specialists. Quality was tested with an Index of item objective congruence (IOC) between 0.67-1.00 for all items.

2) Blood collection and analysis equipment included a tourniquet, dry cotton, alcohol, needle, 3 ml clot blood tube, transpore, and a bag for contaminated trash. Biochemical analysis involved using Ortho Clinical Vitros 350, USA by the enzyme matric method, OCD Drychem. The qualitative analysis laboratory passed International Organization for Standardization (ISO) 15189: 2012 No. 4247/63 and the Thai Ministry of Public Health’s MOPH standard.

### Data collection

1) For the collection of interview questionnaires, the researcher collected the data by interviewing the samples individually in the fuel station areas provided by the station owner. The interviews were conducted after working time and took about 10-15 minutes per person. Interpreting the results of the interviews on the important variables was done as follows:

(i) Job positions variable - The researcher interviewed samples for the main job positions, who were divided into two groups, comprising the I-FDA group and the O-FDA group, based on previous studies both in Thailand [5,26] and internationally [1,4]. The two groups of job positions can be classified into 10 types of positions according to employee operations such as refueling, cashier, loading oil into storage tanks, convenience store, coffee shop, selling in an open building, selling food in a closed building, office staff, general staff, and car washers. Interpretation of the score gave 1 = working in that job position, and 0 = not working in that job position.

(ii) Wearing PPE necessary for the operation of fuel stations – The researcher applied the findings from a study by Alves et al. [18] concerning the use of PPE related to occupational exposure among gasoline station workers. A total of 7 types of PPE were classified, namely glasses, masks (medical mask, carbon, and N95), gloves, long sleeves, long pants covering the ankles, chemical protective clothing, and boots or heels. Interpretation of the score gave 1 = wearing that type of PPE for more than 3 hours per day, and 0 = not wearing or wearing that type of PPE for less than 3 hours per day.

2) Blood sample collection to analyze AChE activity levels was done after each shift. Approximately 3 ml of blood was collected into clot blood tubes and drawn by a medical technician or professional nurse. The collection location was the same as the interview point. After blood collection, the researcher soaked the blood sample in a box containing ice packs to maintain the temperature at 4 degrees Celsius for transport to the laboratory for analysis within the same day as the sample collection. The AChE activity was analyzed according to the Ellman et al. [27]. The results were interpreted according to the Diagnosis Laboratory Center [28] with normal reference values, i.e. females between 4.65 – 10.44 U/ml, and males between 5.9 – 12.22 U/ml. In the study, controls for activities that might affect AChE activity levels, i.e. being a fuel station away from pesticide-treated agriculture, was controlled. Participants in the research project did not have a side job in chemical spraying, and one week prior to blood collection for AChE activity assessment, no waste was burned in their house or accommodation areas.

### Statistics

The quantitative data were tested for distribution with a histogram. The statistics used in the data analysis were divided into two categories: 1) Descriptive statistics (i) Discrete variables including gender, education, alcohol consumption, smoking, work area, job position, and wearing PPE were used for frequency and percentage calculations. (ii) Continuous variables were body mass index, hours worked per day, working hours per day, and serum AChE activity using mean, standard deviation, median, and min-max. 2) Inference statistics (i) Comparative analysis between confounding factors with AChE activity levels using an independent t-test. (ii) Comparative analysis of the proportion of AChE activity between I-FDA and O-FDA groups using the chi-square test and risk estimate odds ratio. (iii) Comparative analysis between job positions and PPE behaviors with AChE activity levels using an independent t-test, at a statistical significance of 0.05.

## Results and Discussion

### Demographic and PPE-wearing behavior

In this study, the majority of fuel station employees were female (68.5%), and the mean age was 30.25±11.01 years, while the mean body mass index (BMI) was 23.63±5.25 kg/m2, single status was 52.5%, and graduated from junior high school was 31.0%. They had an average monthly income of 316.19 ± 115.58 USD (1 USD = 38.12 THB on 29 Sep 2022). The results of PPE-wearing behavior among fuel station operators found that most of the workers wore a disposable or medical mask at 84.0%, followed by wearing white pants covering the ankles and boots or covered heels (80.5 and 49.0%, respectively). The less common PPE-wearing behaviors included the use of N95 masks, full-body protective clothing and protective goggles at 1.0, 2.0, and 5.0 percent, respectively.

Employees at fuel service stations are continuously exposed to mixed organic solvents (OS) for long durations [1,4]. In this study, the samples had an average of 2.44±4.06 years working in a station, with an average working day of 9.05±1.56 hours and an average working week of 6.31±0.47 days. Occupations such as these without proper exposure protection are likely to develop health effects. Several studies have looked at the effects of OS in fuel on neurological symptoms [6,8,29]. However, this study used a different neural marker from previous studies. In other words, the AChE activity marker was measured. AChE is a marker that is sensitive to chemical changes [13], and can affect chronic disease or death [30]. Therefore, the prevention of occupational risk factors is a guideline for occupational health. The study highlights job positions at risk for neurotransmitters and the preventable use of PPE.

### Comparison between confounding factors with serum AChE activity levels

This research study investigates the impact of chemical exposure in the context of fuel-related work environments. Workers within this domain routinely encounter various chemical compounds during their occupational engagements. The selection criteria for participants in this research endeavor involved individuals devoid of chronic neurological or psychiatric conditions, alcohol dependency, or usage of medications affecting the nervous system. However, the study also considered additional potential confounding variables, such as smoking habits, alcohol consumption [31], dietary patterns (including the consumption of agricultural fresh produce, fruits, and foods containing preservatives) [32], medication usage (specifically, anticholinergic drugs like sedatives and anti-nausea agents, dizziness-relieving drugs) [33], and the geographic locations of the fuel stations, categorized into municipalities and districts. The study's findings indicated that individuals who encountered or ingested these elements, or resided near gas stations in urban regions, exhibited AChE activity that did not vary significantly from individuals who had no exposure to these factors. Our findings from the analysis unequivocally establish that the examined confounding factors exert no discernible influence on AChE activity levels (Table 1).

### Comparison of serum AChE activity levels between I-FDA and O-FDA groups

There are many job positions at fuel stations in Thailand. In this study, the main areas of operators were divided into two parts: I-FDA and O-FDA groups. Considering the job positions of work, it was found that there were 43.5% refueling operators and 11.5% cashiers in the I-FDA group area. At some fuel stations, cashiers also performed refueling duties, thus having both job characteristics in the same person. As a result, two types of jobs are done by the same person. For the O-FDA group, there were similar proportions of job positions, namely working in an office, coffee shop, selling in an open building, and convenience store, which were 10.5, 9.5, 9.0, 8.5 and 8.0, respectively. However, less common job positions included car wash workers and selling at food shops in a closed building, which totaled 2.5 and 3.0 %, respectively.

**Figure 1.**
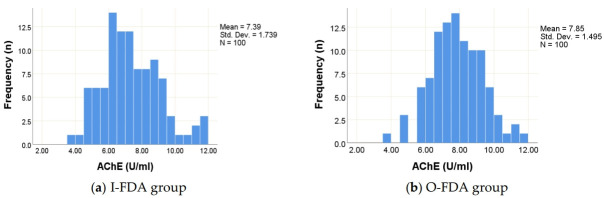
Histogram of serum AChE activity levels for (a) Inside fuel dispenser areas [I-FDA] group and (b) Outside fuel dispenser areas [O-FDA] group

The results showed that the I-FDA group had a lower statistical significance of mean serum AChE activity (7.38 ± 1.73 U/ml) than the O-FDA group (7.85 ± 1.49 U/ml) (*p*<0.05). Both groups had a normal distribution histogram (Figure 1). This study was one of the first reports describing AChE activity data among workers at gas service stations. Therefore, the report of this study indicated the histogram distribution to illustrate the nature of the data. From the analysis, it could be seen that the distribution curve was normal, which could represent populations with similar occupational contexts and was reliable according to the conditions of inferential statistics.

All subjects had serum AChE activity proportions below the criterion by 12.0%. When comparing the difference in the proportion of serum AChE, it was found that the I-FDA group had 2.43 times the risk of abnormal serum AChE activity compared to the O-FDA group (OR=2.43; 95%CI=1.05-5.60) (Table 2).

The I-FDA group had a statistically lower AChE activity than the O-FDA group. In the past, there were still doubts about the OS substance and the level of AChE activity. In animal studies, rats were exposed to OS at chemical concentrations between 0.6-10.0%. The results showed that the levels of AChE activity in the rats' brains decreased. Specifically, acetone and acetonitrile significantly inhibited AChE in all structures [34]. In addition, Olivares-Rubio and Espinosa-Aguirre [35] discussed oil hydrocarbons with AChE activity from a new perspective. Olivares-Rubio and Espinosa-Aguirre studied AChE in fish affected by polycyclic aromatic hydrocarbons (PAHs) contamination from crude oil stains in water. The results of the study showed that the substance benzo[a]pyrene, pyrene and anthracene inhibited AChE activity. However, the study noted that PAHs in oils have low molecular weight, causing changes and/or the stimulation of AChE activity. Thus, it may require concern for experimental in vitro and in vivo designs and applying multivariate statistical and correlation analyses between these pollutants with AChE activity in field studies [35].

Although this study is a field practice, further studies are needed to support these findings to confirm the results. If possible, a research model that measures the amount of AChE activity twice may be formulated in the future, i.e. before work and during work. For example, Sombatsawat et al. [14] studied AChE activity in agriculturists. The first time was measured for AChE activity 30 days before the start of seasonal farming, and the second time was measured 30 days after the first farming. Therefore, decreased AChE activity can be an indicator of insecticide poisoning. However, working at a fuel station is different because there is no long-term break [5]. Therefore, the AChE activity assessment may be done the first time by collecting data before entering the workplace and the second time after working for a period of time to confirm the results.

### Comparison between job positions and PPE wearing with serum AChE activity levels

The results showed that job positions including refuelers and cashiers had lower statistical significance for AChE activity levels than other job positions (*p*<0.05) (AChE activity level for refuelers was 7.40±1.49, while non-refuelers was 7.79±1.49 U/ml, and cashier was 7.05±1.82, while non-cashiers was 7.69±1.5 U/ml). In addition, those who worked in closed buildings had higher AChE activity levels than those who did not (9.15±1.45 & 7.57±1.61 U/ml, respectively). For wearing PPE, it was found that those who wore medical masks for work had higher AChE activity levels than those who did not (8.19±1.61 & 7.51±1.62 U/ml, respectively). Other types of PPE showed no difference in AChE activity levels (Table 3).The analysis of job positions found that refuelers and cashiers had lower AChE activity than those with other job positions. As mentioned above, previous studies of oil chemical exposure and AChE activity were only focused on animals [34,35]. Most of the previous studies in humans involved AChE activity with poisoning from other chemicals, especially organophosphate, carbamate insecticides [14] or heavy metals such as lead [36]. However, Thailand has already eliminated the lead mixture in fuel [37].

It is noteworthy that some pesticides contain OS-based data. Kapeleka et al. [20] found that 34.6% of the substituted benzene used by chemical families of pesticides was used in horticultural production. These chemicals will inhibit AChE activity. Therefore, the measurement of AChE activity was used as a biomarker of toxic exposure [38]. However, this study only took samples for AChE activity analysis once and the fuel compounds contained several chemicals, such as VOCs and PAHs [2]. Therefore, combined studies of multiple compounds should be confirmed [35]. However, the study identified the targets of the risk group as those working closely with fuel injectors, which should be a priority.

However, it is worth noting that those loading fuel into storage tanks at each station worked for a short period of time. Loading fuel from tanker trucks into storage wells was conducted in a closed system where a pipe was connected from trucks to storage wells. The pipe and the hole of the storage wells were similar in size, which could reduce the evaporation gap. In addition, loaders wore respiratory protection while working more than usual because, while loading, the smell of fuel would be pungent. As a result, AChE activity levels were not different from those of other groups.

The results of the study were found to be similar to others. Working in an enclosed building can be a protective factor against the effects of AChE activity. In this study, it was found that working in a closed building resulted in higher AChE activity levels than those who did not. A closed-building food shop in a fuel station is some distance away from the refueling area; each fuel station has a similar store layout. That is, the area near the fuel dispenser area will be a convenience store. Next to it is a coffee shop and food shop located some distance from the fuel station. Increased distance may decrease the concentration of the chemicals. In addition, there was a similar study of hazards from fuel. According to Heibati's study [39], workers exposed to BTEX from petroleum were investigated. Tanker loading workers were more exposed to benzene than office workers, both of which worked in the same work areas. Therefore, it could be seen that staying in an enclosed room with concrete walls could prevent the vapors of substances in fuel. As the enclosed room used a ventilation system in the building by circulating air from inside the room and there was a fan to vent the air to the outside, the concentration of the substances in the enclosed building might decrease more than outside, which was the source of the substances at fuel dispensers. Therefore, the enclosed room could prevent the vapors of substances and reduce the effect on neurotransmitters.

However, it is worth noting that the results indicated similar AChE activity levels in the I-FDA and O-FDA groups and those wearing PPE in which the appropriate protection against vapors of substances in fuel should also be considered, the next study should collect more detailed or frequency data by covering the types of PPE and the duration of wearing.

The food stores in closed buildings at fuel station areas are different from convenience stores in terms of consumer behavior since the food stalls serve main meals. Therefore, there are many users at certain times, such as lunch break or after work, etc., resulting in less frequency of closing and opening doors than convenience stores. In addition, most of the doors used in food stores are push-pull by themselves, which is different from convenience stores that use automatic doors, causing pedestrians to walk through the food stores because the door does not slide open by itself. This makes people working selling food in closed buildings less likely to be exposed to OS and the effects of oil than convenience store employees. In addition, convenience stores have a variety of products to choose from, such as food, snacks, beverages, consumer goods, miscellaneous goods, etc. As a result, there are more users than food stores. In a recent study in Brazil, there was no difference in exposure to benzene in convenience stores and refueling operations [1]. In some chemicals, such as toluene and xylene, exposure was more common in convenience store salespeople than in refueling operations [4]. In this study, the effects of OS on health were investigated. AChE activity was not different in the convenience store and fuel efueling operations.

The main protective factors found in this study on the issue of wearing masks. The results of the study found that those who wear disposable masks at work have higher AChE activity levels than those who did not. Roughly 84.0% of the workers wore masks, which is higher than in previous studies [18,40]. This may be due to the collection of data during the Covid-19 epidemic, resulting in most people using masks more. The medical mask is a mask that is commonly used by workers, probably because they are as accessible and inexpensive as carbon or N95 masks; employees wear these masks more than others, and it can be seen that operators wearing disposable masks feel more comfortable than other masks, so they can be worn for a long time. Therefore, it may be the cause of protection against exposure to chemicals that can affect the level of AChE activity. Consistent with Sirivarasai's study, [41] AChE activity was compared between those who wore PPE and those who did not. Among the general public who have the opportunity to be exposed to organophosphates, it was found that AChE activity was statistically different (*p*<0.05).

On the issue of wearing OS masks, the OSHA agency has recommended using respirators to protect against chemical vapors; masks should have air-purifying respirators or cartridge or filter masks [42]. In practice, however, it can be seen that operators are still unable to use such masks. The results of the research indicated that disposable mask protection can prevent the impact of AChE. Thus, a policy to make masks necessary PPE for workers in fuel station areas is recommended. There are measures to control and monitor the wearing of masks by employees so they get used to it, and create a safe culture at the stations. However, consideration should be given to the suitability of the use of respiratory masks, such as using the right type, the right method, and an affordable price. In the past, the study of the effectiveness of the masks commonly used in 4 types, namely fabric, surgical, and carbon, and an N95 mask for protection against chemical vapors, was done. The results showed that surgical, carbon and N95 masks offered better protection than fabric masks with statistical significance (*p*<0.05) [43]. Therefore, employers should consider using them in practice.

A highlight of this study is the field-based human pilot study of fuel chemical exposure and levels of AChE activity, which were previously suspected and studied in a laboratory and observed among animals contaminated by oil spills. The results of the study support the link between chemicals in fuel and neurotransmitters. However, a limitation of this study was the classification of job positions. In some jobs, there may have been personnel redundancies, such as those among refuelers and cashiers; in a rush hour of refueling, a cashier can help perform the job of pumping fuel if there is a large volume of vehicles coming in for service. The researchers of the current study interviewed only the main job positions. Thus, future research should consider the potential classification error among people performing more than one job description, which may further confirm the findings.

## Conclusions

This study indicated a target group of workers at reduced risk of AChE activity, with the I-FDA group having lower AChE activity than the O-FDA group. From the analysis of job positions, refuelers and cashiers had lower AChE activity than those without these job positions. Working in an enclosed building and wearing a respiratory protective equipment were factors for good protection. In the next study, for the benefit of practical applications, the emphasis should be placed on studying respiratory protective equipment factors, such as the protective efficacy of different masks that can be easily accessed or the sufficient duration of wearing or taking care of a mask to protect health, in order to provide clearer information on preventing exposure to fuel vapors. In addition, at the sources of the substances, especially the refueling points, the installation of a recovery vapor system to reduce contamination in the air in that area and to minimize impact on health should be studied.

## Figures and Tables

**Table 1.** Comparison between confounding factors with serum AChE activity levels

Confounding factors		n (%)	AChE activity levels		t	*p*-value
			Mean	SD		
**Behavior**						data
Smoking	No	132 (66.0)	7.68	1.71	0.804	0.422
	Yes	68 (32.0)	7.49	1.59		
Alcohol drinking	No	109 (54.5)	7.74	1.65	1.149	0.252
	Yes	91 (45.5)	7.47	1.60		
Taking drugs that cause drowsiness or pain relief	No	135 (67.5)	7.50	1.64	-1.448	0.149
	Yes	65 (32.5)	7.86	1.59		
**Dietary**						
Leafy green vegetables	No	62 (31.0)	7.38	1.42	-1.350	0.177
	Yes	138 (69.0)	7.72	1.71		
Tomato	No	101 (50.5)	7.62	1.73	0.037	0.970
	Yes	99 (49.5)	7.61	1.53		
Cauliflower	No	88 (44.0)	7.53	1.65	-0.624	0.534
	Yes	112 (56.0)	7.68	1.62		
Lettuce	No	116 (56.0)	7.66	1.54	0.435	0.664
	Yes	84 (42.0)	7.56	1.75		
Long beans	No	109 (54.5)	7.53	1.54	-0.828	0.409
	Yes	91 (45.5)	7.72	1.73		
Fruit juice box	No	95 (47.5)	7.68	1.57	0.560	0.576
	Yes	105 (52.5)	7.55	1.69		
Prunes or dried fruit	No	151 (75.5)	7.62	1.63	0.146	0.884
	Yes	49 (24.5)	7.59	1.64		
Turmeric	No	160 (80.0)	7.66	1.63	0.767	0.444
	Yes	40 (20.0)	7.44	1.65		
Honey	No	119 (59.5)	7.68	1.58	0.720	0.473
	Yes	81 (40.5)	7.51	1.70		
Durian	No	111 (55.5)	7.57	1.54	-0.448	0.654
	Yes	89 (44.5)	7.67	1.74		
**Location of fuel station**						
Municipal		137 (68.5)	7.63	1.75	0.151	0.880
Non-municipal		63 (31.5)	7.59	1.34		

**Table 2.** Comparison of serum AChE activity levels between I-FDA and O-FDA groups

AChE activity	I-FDA (n=100)	O-FDA (n=100)	Total(n=200)	*χ* ^2^	OR	95%CI
Serum AChE activity						
- Normal, n(%)	83 (83.0%)	93 (93.0%)	176 (88.0%)	4.735	2.43	1.05-5.60
- Abnormal, n(%)	17 (17.0%)	7 (7.0%)	24 (12.0%)			
Mean (U/ml)	7.38	7.85	7.62			
SD	1.73	1.49	1.63			
Median	7.18	7.88	7.53			
Range	3.85-11.94	3.72-11.69	3.72-11.94			

Note: Risk estimate odds ratio by chi-square test

**Table 3.** Comparison between job positions and PPE behaviors with serum AChE activity levels

Job positions and PPE behaviors		n (%)	AChE activity levels		t	*p*-value
			Mean	SD		
**I-FDA**						
Refueler	No	109 (54.5)	7.79	1.49	1.690	0.043*
	Yes	91 (43.5)	7.40	1.77		
Cashier	No	177 (88.5)	7.69	1.56	1.763	0.039*
	Yes	23 (11.5)	7.05	1.82		
Loading fuel into storage tanks	No	191 (95.5)	7.63	1.64	0.567	0.572
	Yes	9 (4.5)	7.31	1.56		
**O-FDA**						
Convenience store staff	No	184 (92.0)	7.59	1.63	-0.878	0.381
	Yes	16 (8.0)	7.96	1.58		
Coffee shop staff	No	183 (91.5)	7.57	1.63	-1.211	0.227
	Yes	17 (8.5)	8.07	1.60		
Shopkeepers in an open building^1^	No	181 (90.5)	7.60	1.61	-0.316	0.752
	Yes	19 (9.5)	7.73	1.84		
Food salesperson in a closed building^2^	No	194 (97.0)	7.57	1.61	-2.365	0.019*
	Yes	6 (3.0)	9.15	1.45		
General worker	No	188 (94.0)	7.64	1.64	0.763	0.447
	Yes	12 (6.0)	7.27	1.43		
Car washer	No	195 (97.5)	7.61	1.63	-0.152	0.879
	Yes	5 (2.5)	7.73	1.70		
Office worker	No	179 (89.5)	7.61	1.68	-0.224	0.823
	Yes	21 (10.5)	7.69	1.17		
**PPE wearing**						
Goggles	No	190 (95.0)	7.61	1.65	-0.152	0.879
	Yes	10 (5.0)	7.69	1.12		
Medical mask	No	32 (16.0)	7.51	1.62	-2.189	0.030*
	Yes	168 (84.0)	8.19	1.61		
Carbon mask	No	126 (63.0)	7.39	1.33	-1.607	0.110
	Yes	74 (37.0)	7.75	1.77		
N 95 mask	No	198 (99.0)	7.30	1.14	0.549	0.584
	Yes	2 (1.0)	7.63	1.65		
Gloves	No	158 (79.0)	7.55	1.69	-1.012	0.313
	Yes	42 (21.0)	7.84	1.36		
Boots or covered heels	No	102 (51.0)	7.72	1.53	0.945	0.346
	Yes	98 (49.0)	7.50	1.73		
Chemical protective clothing	No	196 (98.0)	7.62	1.65	0.244	0.808
	Yes	4 (2.0)	7.42	0.42		
Long-sleeved shirts	No	142 (71.0)	7.55	1.59	-0.865	0.388
	Yes	58 (29.0)	7.77	1.72		
Trousers	No	39 (19.5)	7.83	1.70	0.908	0.365
	Yes	161 (80.5)	7.56	1.61		

1 Shopkeepers in open buildings are samples who sell in buildings with no wall on at least one side, such as for selling fruit, fried food and beverages, etc.,

2 Food salesmen in closed buildings are samples who sell in closed buildings all the time, such as restaurants, using air conditioning systems.
